# Light-enhanced cognitive behavioural therapy for sleep and fatigue: study protocol for a randomised controlled trial during chemotherapy for breast cancer

**DOI:** 10.1186/s13063-020-4196-4

**Published:** 2020-03-27

**Authors:** Helena R. Bean, Lesley Stafford, Ruth Little, Justine Diggens, Maria Ftanou, Marliese Alexander, Prudence A. Francis, Bei Bei, Joshua F. Wiley

**Affiliations:** 1 School of Psychological Sciences and Turner Institute for Brain and Mental Health, Monash University, 18 Innovation Walk, Melbourne, VIC 3800 Australia; 2 Centre for Women’s Mental Health, Royal Women’s Hospital, Melbourne, Australia; 3 Melbourne School of Psychological Sciences, University of Melbourne, Melbourne, Australia; 4 Peter MacCallum Cancer Centre, Melbourne, Australia; 5 Melbourne School of Population and Global Health, University of Melbourne, Melbourne, Australia; 6 Pharmacy Department, Peter MacCallum Cancer Centre, Melbourne, Australia; 7 Sir Peter MacCallum Department of Oncology, University of Melbourne, Parkville, Australia

**Keywords:** Light therapy, Cognitive behavioural therapy, Sleep, Insomnia, Fatigue, Randomised controlled trial, Chemotherapy, Breast cancer

## Abstract

**Background:**

Women with breast cancer experience a significantly higher prevalence of sleep disturbance and insomnia than the general population. The experience of persistent sleep disturbance places these women at a higher risk of psychological and physical morbidity and a reduced quality of life. Treatment for sleep in this population is not part of routine care and is often managed inadequately. This randomised controlled trial will examine the combined effects of cognitive behavioural therapy (CBT) and bright light therapy (BLT) on the symptoms of insomnia, fatigue and mental health.

**Method/design:**

Women diagnosed with breast cancer who receive intravenous chemotherapy treatment at a quaternary referral metropolitan cancer centre in Melbourne, Australia, will be recruited. Recruitment will occur after diagnosis and prior to completion of chemotherapy. Eligible women will be randomised to the combined CBT and BLT intervention (CBT+) or relaxation audio-enhanced treatment as usual (TAU+). The CBT+ group will receive one face-to-face session on sleep strategies, one subsequent telephone call, and seven email packages containing CBT-based information and strategies. CBT+ participants will also wear Luminette® light glasses for 20 min each morning for the 6-week duration of the intervention. Women in TAU+ will receive two relaxation audio tracks via email. Outcomes will be measured at multiple points throughout the 6 weeks. Primary outcomes will be symptoms of insomnia and sleep efficiency, measured using the Insomnia Severity Index and a self-reported sleep diary. Secondary outcomes include objective measures of sleep assessed using the ActiGraph wGT3X-BT, and sleep-related complaints, fatigue and mental health, all assessed using the Patient-Reported Outcomes Measurement Information System (PROMIS). Data will also be collected on potential treatment moderators and mechanisms and adherence to treatment. There will be 3-month follow-up measurements of insomnia symptoms, fatigue, sleep-related impairment, sleep disturbance, depression and anxiety.

**Discussion:**

This is the first randomised controlled trial to combine CBT and BLT for the treatment of sleep disturbance in women with breast cancer. This novel design addresses the multiple causal factors for sleep complaints in this population. Results from this trial will advance knowledge in this field and may have important clinical implications for how best to treat sleep disturbance and insomnia in this population. If effective, the largely email-based format of the intervention would allow for relatively easy translation.

**Trial Registration:**

Australian New Zealand Clinical Trials Registry (ANZCTR), ACTRN12618001255279. Retrospectively registered on 25 July 2018.

## Background

Breast cancer (BC) is the most common cancer in women, with an estimated 19,371 new diagnoses in 2019 in Australia [1]. Among women with BC, a high prevalence of sleep disturbance and insomnia are reported before, during and after treatment for BC in both early-stage and metastatic cancer [2]. Prevalence estimates for insomnia in individuals with BC range from 30% to 60% [3], 3–6 times the prevalence in the general population [4]. Furthermore, up to 87% of women with BC report sleep disturbances that may not meet the clinical criteria for insomnia [5], yet still yield negative consequences including a higher risk of psychological and physical morbidity and a reduced quality of life [6, 7]. Sleep disturbance in women with BC is linked with poor physical recovery, an increased likelihood of BC recurrence, impaired cognitive functioning, decreased work productivity, medication misuse and abuse, and poorer interpersonal relationships [8, 9]. The increased risk of recurrence is of particular concern to both individuals and their loved ones, and places additional costs on the health care system. Despite most women who are treated for BC experiencing significant sleep disturbance and elevated rates of insomnia, sleep treatment is not standard and sleep disturbance or insomnia are commonly managed inadequately, in part due to standard treatments being time and resource intensive.

### Causes of sleep disturbance among women with BC

The high prevalence of insomnia and sleep disturbance experienced by people with cancer has been attributed to cancer treatments and their side effects, psychosocial factors such as anxiety, stress and depression, and circadian disruption. These are discussed in the following sections.

#### BC treatments and sleep disturbance

The most common treatments for BC include surgery, radiotherapy, chemotherapy and hormonal therapy. Physical symptoms caused by treatment (for example, nausea, hot flushes, night sweats, urinary frequency, pain, changes in body image and skin reactions) are recognised as the main treatment-related factors perpetuating sleep disturbance [5]*.* Many of these symptoms are side effects of anticancer or supportive care medications. Thus, it is not surprising that a recent systematic review investigating the impact of treatment for BC on sleep found that women undergoing chemotherapy and radiotherapy showed the greatest sleep impairments in comparison to other BC treatment regimens [10]. Previously, Palesh and colleagues [5] found the prevalence of sleep disturbance among cancer patients to be highest during chemotherapy treatment, with up to two-thirds of patients undergoing chemotherapy reporting impaired sleep. Corticosteroids, such as dexamethasone, are commonly used in the days surrounding a chemotherapy cycle and acutely increase alertness and insomnia [11], representing one reason why sleep often is disrupted during this time. Surgical treatments also impair sleep due to disruption during hospitalisation, as well as postoperative pain and inflammatory responses. However, sleep disturbance following surgery is less severe than the disturbance associated with chemotherapy, hormonal therapy and radiotherapy [10].

#### Psychosocial factors and sleep disturbance

BC diagnosis and treatment are associated with psychological challenges [12], including elevated levels of life stress, depression and anxiety [13], which negatively impact sleep [3]. Common experiences include intrusive thoughts, worry and rumination surrounding diagnosis, treatment and prognosis [14]. Anxiety and stress are linked with sleep disturbance among people with cancer during both active treatment [15, 16] and survivorship [2]. Depression is common, with research showing that over 16% of women with BC meet criteria for major depressive disorder and nearly 40% have persistently elevated depression symptoms in the year after diagnosis [17]. Sleep disturbance is a recognised symptom of depression [18] and depression predicts sleep disturbance among women with metastatic BC [13].

#### Circadian disruption and sleep disturbance

Sleep, wake and other behavioural and physiological processes are typically entrained to zeitgebers (environmental time cues), with the most prominent being the 24-h cycle of light and dark. Compared to healthy controls, people with BC display evidence of more disrupted circadian rhythms, including decreased amplitude of rest activity rhythms [19–22]. Chemotherapy has been implicated as a key contributor to these disruptions [23, 24]. In women with BC, 24-h sleep–wake rhythms worsens during chemotherapy [25]. Despite some brief recovery after each chemotherapy cycle, sleep–wake rhythms appear to become increasingly worse over multiple cycles [25]. Therefore, ongoing chemotherapy may be partly responsible for the deterioration of circadian rhythms among women with BC.

Disruptions to circadian rhythms are implicated in cancer-related sleep disturbance [26, 27], depressive symptoms and worse overall quality of life [3]. Research also demonstrates associations between circadian disruption and cancer incidence and progression [26]. Mormont and colleagues [28] found that people with cancer exhibiting dysregulated circadian rhythms were five times more likely to die within 2 years compared to those with more distinguishable circadian rhythms. Core circadian genes may be important in tissue homeostasis and tumorigenesis such that the disruption of circadian rhythms accelerates tumour progression. It has been argued that restoring circadian rhythms through sleep interventions could improve prognosis [27].

### Treating sleep in cancer

#### Cognitive behavioural therapy

Cognitive behavioural therapy (CBT) for insomnia is well established as an effective treatment for insomnia, with efficacy comparable to sleep medication in the short term and superior in the long term [29]. Individuals with insomnia—whether or not it is comorbid with a medical condition such as cancer—typically exhibit cognitive and physiological hyperarousal, demonstrate attentional biases, and endorse problematic, sleep-related beliefs [3]. Typically, CBT is delivered as a multi-component intervention to address these inter-related aspects of insomnia and may include sleep restriction, stimulus control, sleep hygiene, cognitive restructuring and relaxation training. A systematic review of CBT in people with cancer found that CBT has larger effects on treating both insomnia and sleep disturbance than pharmacotherapy, medication placebo, relaxation therapy, sleep hygiene education and ‘treatment as usual’. Critically, CBT has durable effects long after treatment completion [3]. Even when factors outside of an individual’s control (for example, medications and physical symptoms) interfere with sleep, CBT-based strategies can improve sleep. This is because CBT increases sleep drive, extinguishes conditioned arousal, and focuses on altering maladaptive behaviours and cognitions that individuals with insomnia adopt. Growing evidence demonstrates that the insomnia and sleep disturbances that present comorbid with psychiatric or medical disorders are responsive to CBT [30]. Importantly, CBT improves subjective sleep outcomes in people with cancer [3].

Traditionally, CBT for insomnia (CBT-I) is delivered in one hour face-to-face sessions with a trained clinician. Despite the evidence for its efficacy, the demanding requirements of CBT for both individuals and clinicians limit its accessibility, particularly given the medical and symptom burden people with cancer already face. Consequently, a small number of studies have trialled condensed versions of CBT in this population. The efficacy of a 9-week internet-based CBT programme was examined in a sample of cancer survivors. The study reported that those receiving the intervention demonstrated significantly greater improvements in insomnia severity, sleep efficiency, and sleep quality when compared to a control group [31]. In people with BC, Savard and colleagues [32] compared video-based CBT, professionally administered CBT-I, and a no-treatment group. Professionally administered CBT resulted in the greatest sleep improvements, but video CBT showed significant sleep improvements compared to the no-treatment group. These studies provide evidence for the efficacy of CBT in people with cancer, even when delivered in a reduced but more accessible format.

#### Light therapy

Bright light is one of the strongest cues for circadian timing; in addition, it has an alerting effect and can reduce fatigue [33], a common symptom particularly following chemotherapy [34]. Bright light therapy (BLT) is a simple form of treatment with relatively low costs, and its efficacy in treating circadian disruption has been well documented [33, 35–37]. Although BLT is effective in a range of populations presenting with insomnia, disturbed sleep or fatigue, there are no guidelines to date for the use of BLT for specific sleep complaints or clinical populations. There is consensus in research regarding the timing of BLT treatment [33]. Exposure to BLT around habitual waking time may shift the circadian rhythm earlier, whereas BLT in the biological evening could shift the circadian rhythm later. BLT has its greatest effect on circadian rhythms immediately before or after the temperature nadir (the lowest point the core body temperature falls to overnight, typically between 4 and 6 a.m.) [33]. Thus, BLT in the morning should ideally occur early, without forcing individuals to wake too early resulting in daytime sleepiness.

Despite the evidence for circadian disruption among women with BC and the efficacy of BLT, few studies have investigated BLT in this population. Ancoli-Israel and colleagues [38] tested BLT to prevent fatigue in women undergoing chemotherapy. In their study, the BLT protocol consisted of placing a 10,000 lux light box approximately 45 cm from the participant’s head within a 45° angle from the midline of the visual field. The light box was used for 30 min each morning on awakening throughout the first four cycles of chemotherapy. This intervention was effective in preventing women from experiencing fatigue during chemotherapy [38]. In the same study, BLT also protected women from circadian disruption during chemotherapy [26]. Redd and colleagues [39] utilised the same BLT protocol for 4 weeks in a sample of BC survivors, and found that BLT significantly improved cancer-related fatigue. Similar findings were previously reported by Liu and colleagues [40]. However, despite the promising evidence, there is little evidence on whether BLT improves sleep, not just fatigue, during chemotherapy for BC.

### Moderators

Although CBT and BLT are effective interventions, not all people respond to treatment, highlighting the need to identify moderators of treatment response. The current study focuses on two categories of moderators: 1) factors that influence treatment engagement such as patient expectations; and 2) factors around the cause of sleep disruption, such as cancer and supportive care treatments and physical symptoms. Perceived treatment credibility and expectations of therapeutic outcomes may act together to influence treatment engagement and adherence. Research shows that positive expectancies are associated with greater adherence and better treatment outcomes to CBT [41].

Pain is a frequently identified contributor to insomnia, with prevalence rates between 33% and 52% for women with non-metastatic BC, with rates increasing to 56–68% among women with metastatic BC [13]. Although relaxation components of CBT may reduce pain in people with cancer [42], neither CBT nor BLT target pain directly and thus may be less effective in people with severe or persistent pain. An individual’s sleep chronotype also may moderate the efficacy of sleep intervention. Sleep chronotype refers to the timing of the sleep–wake cycle that is influenced by endogenous (circadian) and exogenous (work/social schedule) factors. It is the propensity for morningness (waking and sleeping earlier in the 24-h cycle) or eveningness (waking and sleeping later). Individuals with evening chronotypes tend to experience increased sleep problems in addition to a greater degree of irregularity in their sleep–wake cycle and greater psychiatric distress [43]. Although CBT improves sleep regardless of chronotype [44], BLT is more effective in individuals with good sleep hygiene and regular sleep timing, both traits of people with morning chronotypes [33]. Conversely, people with morning chronotypes may already be exposed to BLT naturally, so that BLT may be more effective for people with an evening chronotype. Chronotype may therefore moderate the therapeutic effects of light therapy among women with BC.

### Mechanisms

The CBT+ intervention is based on CBT-I and includes the fundamental components of stimulus control, sleep restriction, cognitive restructuring and sleep hygiene. These components are intended to target specific mechanisms of insomnia. As this is a reduced version of traditional CBT and in an understudied population, it is important to examine whether it acts on the same expected mechanisms. Understanding the mechanisms through which the CBT+ programme impacts primary and secondary outcomes also may provide important clinical implications for how best to treat poor sleep in this population.

Individuals with insomnia experience increased worry and rumination and often develop distorted or unhelpful beliefs about sleep [45]. Among women with BC, experiences of anxiety, rumination and intrusive thoughts are particularly common [14]. The cognitive restructuring and psychoeducation components of the CBT+ programme aim to help participants manage night-time worry and shift unhelpful beliefs and attitudes toward sleep. Measuring change in participant’s dysfunctional beliefs and attitudes toward sleep, and their rumination and intrusive thoughts before bed, will provide insight into how changes in these variables may be associated with sleep outcomes.

Hyperarousal also is recognised as a central factor that perpetuates insomnia [46]. When there are repeated associations between poor sleep and the bedroom environment, conditioned arousal occurs, and the bed can become a stimulus for heightened arousal. Cognitive patterns of rumination and anxiety in bed can also contribute to pre-sleep arousal. Stimulus control, relaxation, and cognitive restructuring components of the CBT+ programme can all lead to a reduction in pre-sleep arousal.

Stress has been identified as a common precipitating factor in the development of insomnia. However, the degree to which individuals experience sleep disturbance when exposed to stressors can vary depending on one’s vulnerability to stress-related insomnia [47]. BC diagnosis and treatment are major stressors and those women with a greater vulnerability to insomnia under stress may be at a higher risk of future insomnia. Measuring women’s vulnerability to insomnia under stress throughout the intervention may provide useful information on whether women at greater risk of insomnia development are more or less responsive to the CBT+ programme.

Given the multi-faceted nature and causes of sleep disturbance in women with BC, sleep treatment should target a range of common causes yet be brief and low cost as most people with cancer experience sleep disturbance and urgently need better symptom management.

To address these needs, we designed an innovative, low-cost intervention combining condensed CBT and BLT (CBT+). Together, these therapies address common behavioural, psychological and physiological causes of sleep disturbance. Furthermore, CBT+ addresses the night-time sleep disturbance and the daytime fatigue that are both prevalent in women with BC. In this randomised controlled trial, we evaluate the efficacy of CBT+ versus treatment as usual enhanced with relaxation audio (TAU+) in women being treated with chemotherapy for BC.

### Aims and hypotheses

#### Aim 1

Aim 1 is to test the efficacy of CBT+ compared to TAU+ for improving symptoms of sleep disturbance. It is hypothesised that participants receiving CBT+ will show improvements on measures of sleep disturbance compared to participants receiving TAU+.

#### Aim 2

Aim 2 is to test the efficacy of CBT+ versus TAU+ on psychological outcomes. It is hypothesised that participants receiving CBT+ will experience a decrease in symptoms of depression and anxiety compared to participants receiving TAU+.

#### Aim 3

Aim 3 is to explore potential mechanisms of CBT+ and moderators of the intervention efficacy. Multiple factors may moderate the efficacy of CBT+ including pain, chronotype, perceived credibility and expectations of the intervention, and adherence to the intervention protocol. Given the lack of knowledge surrounding the impact of these potential moderators in the BC population, this study aims to explore whether any of these moderators has a significant effect on intervention outcomes. Furthermore, this study aims to provide insight into the mechanisms through which CBT+ may improve sleep in women with BC. Based on the factors contributing to sleep disturbance that are targeted by CBT+, the following potential mechanisms will be investigated: pre-sleep arousal, beliefs and attitudes about sleep, vulnerability to insomnia under stress, and rumination and intrusive thoughts before bed.

## Method

### Study design

This is a randomised, two-group, parallel, non-blinded, controlled, single-centre, superiority trial. One group will receive combined CBT plus BLT (CBT+). The other group will receive treatment as usual enhanced with a relaxation audio programme serving as an active control (TAU+). All participants will be involved in the trial for the 6-week duration. Measures will be taken at baseline, at the mid-point of the intervention (3 weeks) and post-treatment (6 weeks). A follow-up assessment will occur at 3 months to evaluate any sustained effects of the intervention on primary and secondary outcomes. This trial and protocol adhere to SPIRIT [48] and TIDieR [49] recommendations.

### Recruitment and consent

Women diagnosed with BC who receive intravenous chemotherapy treatment at the Peter MacCallum Cancer Centre (PMCC) in Melbourne, Australia, will be recruited after diagnosis and prior to the completion of chemotherapy. We have taken several steps to ensure we achieve adequate recruitment and enrolment. The PMCC pharmacy department will generate a report of potentially eligible women identified through their chemotherapy chart. The research team will attend the Chemotherapy Day Unit when each eligible woman has a scheduled appointment and approach women in person, which encourages higher enrolment. Due to the stress associated with BC treatment and the commencement of chemotherapy, women will not be approached during their chemotherapy education session or at their first chemotherapy appointment. Potentially eligible women who show an interest in participating will be provided with information about the study. Should they choose to participate, written informed consent will be obtained. After consenting, women will complete further screening assessments to determine whether they are eligible to participate. We have taken steps to help retention and completion of follow-up. The CBT+ condition was carefully designed to minimise burden (see section below on CBT+ intervention). In the TAU+ condition, we include a mid-point telephone call and emails to maintain engagement.

### Screening

Further eligibility will be assessed via structured interviews designed to detect sleep disorders or severe psychiatric disorders. Sections of the Mini International Neuropsychiatric Interview (MINI) [50] will be administered, including those relating to major depressive disorder (module A), generalised anxiety disorder (module N), manic and hypomanic episodes (module C), alcohol dependence and abuse (module I), substance dependence and abuse (module J), current post-traumatic stress disorder not related to cancer experiences (module H), and psychotic disorders (module K). The Duke Structured Interview for Sleep Disorders [51] will be administered in its entirety. A detailed risk management protocol was used to ensure that if previously unidentified disorders were found, women were referred to psychology, psychiatry or sleep clinics, as appropriate.

Inclusion criteria are: women diagnosed with any stage of primary BC; over 18 years of age; receiving intravenous chemotherapy, with or without radiotherapy, during the study period; able to provide informed consent; able to understand and speak English; able to regularly receive and access emails; able and willing to wear light therapy glasses.

Exclusion criteria are: male; receiving neo-adjuvant chemotherapy; diagnosed with a severe psychiatric disorder or severe substance use disorder as identified through the MINI; having a history of migraines. Individuals with very advanced sleep timing, defined as a habitual bedtime before 8 p.m. and rise time before 4 a.m. or delayed sleep timing defined as habitual bedtime after 3 a.m. and rise time after 11 a.m., or irregular or non-24 sleep and wake pattern will be excluded, based on the Duke structured sleep interview.

It is possible that eligible participants will be receiving additional treatment for sleep complaints (for example, psychological treatment, prescribed sleep medication, herbal remedies, and so forth). These participants will not be excluded; however, this information will be obtained via self-reports and medical record extraction and will be adjusted for in sensitivity analyses. Involvement is voluntary, and participants may withdraw from the study at any time. Involvement will not impact participants’ care and their medical treatment will continue as usual.

### Randomisation

Eligible participants will be randomised into the CBT+ or TAU+ group using a complete randomisation scheme generated in advance. Specifically, block sizes of variable size (4, 6 or 8) will be used. Random seeds will be generated to ensure allocation concealment and prevent pre-guessing of the allocation sequence at the end of each block. Randomisation will be stratified by baseline Insomnia Severity Index (ISI) scores (≤7, ≥8) and cancer stage (≤2, ≥3). The randomisation scheme was generated and setup in REDCap (Research Electronic Data Capture) by a member of the research staff (BB) who is not involved in recruitment or delivery of intervention and is not the principal investigator. All participants will receive a random identification number, which will be used to link data from the different assessments. All direct identifiers (for example, name, address and telephone numbers) will be stored separately.

#### CBT+ intervention

The CBT+ intervention involves seven time points when materials are delivered across 6 weeks. The intervention will be delivered via: one face-to-face session of up to 75 min, to be conducted at the PMCC in the Chemotherapy Day Unit; one telephone call lasting approximately 20 min; seven emails (one per week that take approximately 15–20 min to read); and one brief telephone call (2–5 min) at the end of the intervention to ask about adverse events.

Intervention components delivered via email use the online software Mailchimp, which provides professional email templates and automates the timing of intervention delivery.

##### Cognitive behavioural therapy for insomnia

The intervention materials for the CBT component were adapted from the *Cognitive behavioural treatment of insomnia: a session by session guide* [52]. Core components of the intervention content include:

- General information and skills for better sleep (for example, sleep hygiene, relaxation and mindfulness exercises, dealing with night-time worries)
- Fostering healthy attitudes and expectations about sleep following cancer diagnosis and during treatment
- Managing sleep challenges specific to cancer patients (for example, physical discomfort, pain, daytime consequences of poor sleep)
- Identifying and managing symptoms of insomnia (for example, self-monitoring, stimulus control, sleep scheduling, bed restriction)

The CBT face-to-face session will be delivered by HRB, a provisional psychologist and doctoral graduate student who has completed >450 h of clinical client contact and an additional 40 h of specific CBT-I training. HRB will be supervised by senior clinical psychologists JD and BB.

##### Light therapy individualised protocol

The light therapy component will consist of daily use of light glasses for the 6-week duration of the intervention. Participants will receive a pair of Luminette® glasses with light intensity locked at 1500 lux and will be instructed to wear them for 20–30 min after awakening each morning. The specific timing of use will be established with each participant during the face-to-face session, and will be based on their individual sleep–wake timing and chronotype, accounting for early awakenings. A written light therapy manual covers the key components to be discussed in the face-to-face session.

##### Minimising participant burden

The intervention was designed to deliver effective results while being of minimal burden. Given that women with BC already have an intensive treatment regimen, the design of the intervention was carefully considered, weighing the physical and psychological benefits with the effort and time associated with each intervention component. The face-to-face session will be scheduled around participants’ existing appointments to reduce the burden of travel. All other aspects of the intervention can be completed in the comfort of the participants’ own homes. The light glasses are comfortable, easy to wear, and can be worn over prescription eyeglasses. Outside of morning showers, vigorous exercise, or driving, the light glasses are not likely to disrupt participants’ usual morning routines. Wearing the light glasses will not impair participants’ ability to move around and undertake any domestic or work-related responsibilities (such as preparing and consuming food, household cleaning, reading, writing or typing). Furthermore, the intervention is economical from the perspective of the health care system as it is relatively simple for clinicians to deliver and entails minimal financial expense and burden of time. These are important considerations when evaluating whether the intervention, if effective, could be implemented into routine care.

#### TAU+ intervention

The control group will receive two emails containing web links to relaxation audio tracks. These emails will be received during the first and third weeks of the intervention. The first relaxation audio consists of abdominal breathing strategies that may assist in calming the mind and relaxing the body. The second audio consists of a progressive muscle relaxation. Both audio tracks are approximately 15 min long and participants are instructed to listen to these audio tracks whenever they feel it could be beneficial. These relaxation tracks were developed by the Australian Cancer Council to assist people in coping with a cancer diagnosis. The audio tracks do not contain any sleep-specific information; instead, they include general relaxation strategies that can be used by participants at any time during the day or night. The control group will also receive a brief telephone call (15 min) during the fourth week of their study to check in with participants and respond to any queries or complaints. After the 3-month follow-up assessment, women in TAU+ will be offered the seven CBT+ email packages. Women are informed during consent that they will be randomised and, if randomised to TAU+, will receive the CBT+ materials after their final follow-up assessment.

Relaxation audio was chosen as a control so that the efficacy of the intervention can be compared to a general relaxation paradigm that may offer benefits for wellbeing and stress reduction but does not specifically target sleep. Furthermore, an active control will reduce the influence of placebo or expectancy effects and ensure that all women who participate in the study receive some effective support. The number of times TAU+ participants open relaxation emails and click on relaxation audio links will be monitored to evaluate participant engagement with the programme. Notably, both groups continue treatment as usual, including any standard management of sleep symptoms from their oncology team or other health care providers.

### Assessments and outcomes

Table 1 displays the measurements used in this study and the time points at which they will be administered. Screening interviews will be conducted in person during recruitment prior to randomisation. Baseline (week 0), mid-treatment (week 3), post-treatment (week 6), and 3-month follow-up (week 12) questionnaires with self-report measures will be completed online by emailing women a link to a survey on Qualtrics, or via telephone for women who prefer not to complete questionnaires online. Questionnaires completed via Qualtrics will be considered valid if they are completed within ±1 week of the planned time.

**Table 1:**
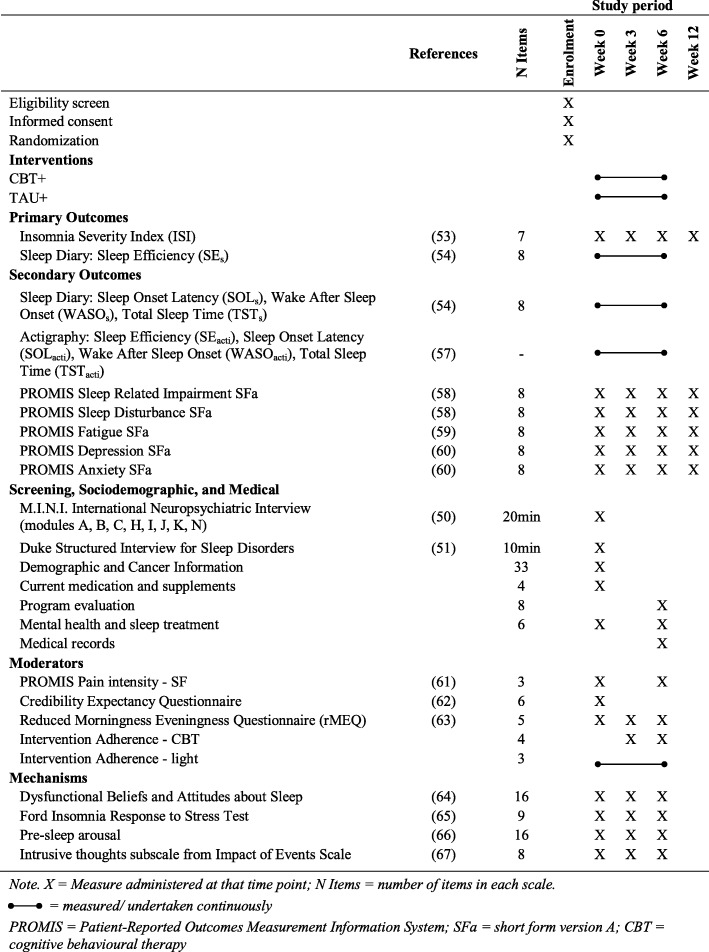
Assessment measures used in the CBT+ study

X indicates measure administered at that time point; N items indicates the number of items in each scale; a continuous line indicates measured/undertaken continuously

*CBT+* combined cognitive behavioural therapy and bright light therapy, *PROMIS* Patient-Reported Outcomes Measurement Information System, *TAU+* treatment as usual plus relaxation audios

During the intervention phase, sleep will be assessed continuously via daily sleep diaries and actigraphy. Sleep diaries will be provided to all women in a paper and pencil format only. The actigraphy watch (ActiGraph wGT3X-BT) is of similar size to a regular wristwatch and will be worn by women continuously during the 6-week intervention. For women in the CBT+ group, the sleep diary also will assess daily light therapy adherence. Prior to commencing the intervention, women will be supplied with six diaries, one for each week of the intervention, and six pre-paid addressed envelopes so that at the end of each week women can post that week’s diary to the research team. Outcome assessment is not blinded as all outcomes (except for sleep based on actigraphy) are patient-reported outcomes.

#### Audio recording

All face-to-face and telephone contacts will be recorded via dictaphone for quality control. Audio recordings will be stored securely with password protection. Written informed consent from participants will be sought before obtaining audio recordings.

#### Primary outcome measures

There are two primary outcomes: changes from baseline to post-intervention in the ISI [53], the gold standard for assessing insomnia symptoms, and self-reported sleep efficiency (SE_s_)_._ SE_s_ will be obtained from the daily sleep diary based on the consensus sleep diary [54]. SE_s_ is calculated using the ratio of self-reported total sleep time divided by the total time spent in bed for sleep. Outcomes will be measured by observing the change in participants’ average SE_s_ between the first week and the last week of the intervention. SE_s_ is commonly used as an indicator of progress in insomnia treatment as high sleep efficiency scores indicate consolidated sleep [55–57].

#### Secondary outcome measures

Secondary outcomes will include additional dimensions of sleep behaviours assessed via self-reported sleep diaries and actigraphy, and symptoms of fatigue, depression and anxiety. Sleep-onset latency, wake after sleep onset and total sleep time will be derived from the sleep diaries and the ActiGraph. The ActiGraph wGT3X-BT records continuous activity information using a three-axis accelerometer along with ambient light. Using the activity data, the wGT3X-BT provides estimates for sleep timing (bed time and rise time), sleep duration (time in bed and total sleep time), and sleep quality (sleep efficiency, sleep-onset latency, wake after sleep onset). Actigraphy data scoring will follow standard protocol, integrating estimates from the automated scoring algorithm along with ambient light, and sleep diary reports of bed and rise time. Actigraphy sleep assessments are time and date stamped so that they can be matched with the self-report sleep diary measures. In sleep studies it is common to evaluate the impact of interventions on multiple behavioural dimensions of sleep as not all dimensions may change. Furthermore, it is helpful to supplement self-reported sleep with an objective measure (actigraphy). Evidence suggests that, often, there is only modest agreement between sleep diary and actigraphy, suggesting that these two measures capture unique information and are not redundant [58].

The Patient-Reported Outcomes Measurement Information System (PROMIS) scales will be used to assess other secondary outcomes including sleep-related impairment and sleep disturbance [59], fatigue [60], and mental health measures of depression and anxiety [61].

#### Treatment moderator and mechanism measures

Potential treatment moderators will be measured as follows. The PROMIS pain intensity short-form scale will be used to assess pain [62]. The six-item Credibility Expectancy Questionnaire [63, 64] will be used to assess participants’ perceived credibility and expectancy of treatment. Chronotype will be assessed using five self-rated items in the reduced Morningness and Eveningness Questionnaire. Treatment adherence for the CBT+ and TAU+ control relaxation components will be measured via self-report questions at the mid-point and end of treatment, assessing the frequency of use and usefulness of strategies. The number of times that participants in the CBT+ and TAU+ groups open emails and click on email links will also be monitored. For the CBT+ group, adherence to light glasses use will be monitored daily and documented as part of participants’ sleep diaries.

The following potential mechanisms of treatment efficacy will also be measured. Beliefs and attitudes about sleep will be measured using the average of 16 self-rated items from the Dysfunctional Beliefs and Attitudes about Sleep Scale and Pre-sleep Arousal Scale [65]. Vulnerability to insomnia under stress will be assessed using the nine-item Ford Insomnia Response to Stress Test [66]. Pre-sleep arousal will be assessed with the Pre-Sleep Arousal Scale [67]. Intrusive thoughts will be measured via the eight-item Intrusive Thoughts subscale of the Impact of Events Scale [68].

### Sample size and power

Based on previous studies of CBT for insomnia and sleep in people with cancer and BLT [69–74] we expect a moderate to large effect size for both primary outcomes. Given that our combined CBT+ targets multiple and different mechanisms of change, we expect larger effects than shown in previous studies utilising only CBT or BLT. Specifically, we expect a standardised mean difference of *d* = 0.70, corresponding to a medium-to-large between-group effect at the post-treatment assessment.

**Fig. 1:**
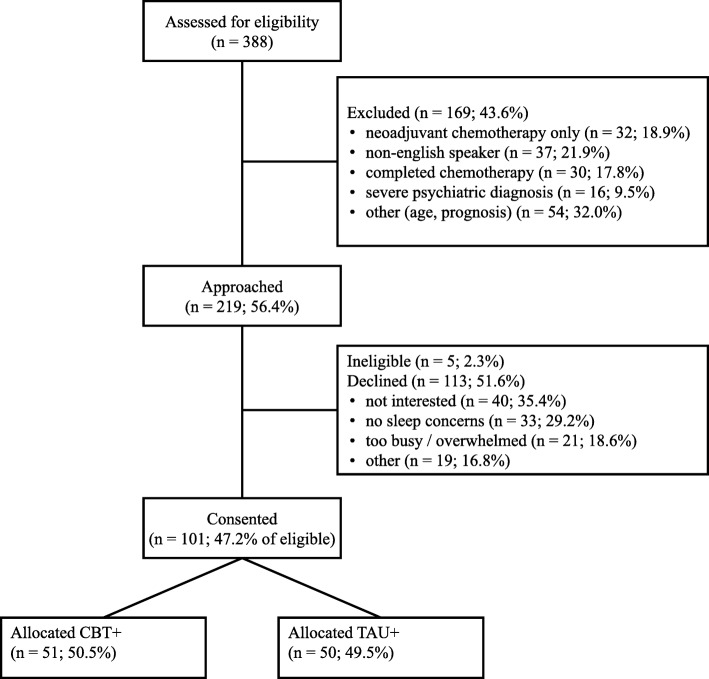
CONSORT participant flow chart of recruitment. CBT+ combined cognitive behavioural therapy and bright light therapy, TAU+ treatment as usual plus relaxation audios

Power analyses based on an independent samples *t* test showed that a total of 70 women will provide >80% power to detect a standardised mean difference of 0.70 at post-treatment, assuming α = 0.05 and equal variance between groups. Power analyses are not readily available for latent growth models (LGMs) and require complex simulations. Therefore, we chose power analysis based off a *t* test as a conservative estimate as, due to randomisation, no baseline differences between conditions are expected so the difference in the slopes from LGMs between treatment and control will be based on post-treatment mean differences but should have lower variability because of removing variability due to individual differences at baseline. Thus, we anticipate the LGMs will have more power than a *t* test based on post-treatment scores and that our power estimates are conservative, although final power will depend on the difference, variability, and correlations in data over time, which are unknown. We will continue recruitment until we reach 70 completers. Progress to date is shown in Fig. 1.

### Statistical analyses

Primary analyses will be conducted on an intention-to-treat basis. Thus, results for all randomised women will be analysed in the group to which they were assigned, regardless of protocol violations. The only exception to this will occur if participants withhold or withdraw consent to use their data in the analysis. Statistical analyses will be conducted in R (data management, graphs, preliminary analyses) and MPlus using MplusAutomation [75]. As a preliminary step, data will be assessed for outliers and skew and, where appropriate, transformed or winsorised. Prior to primary analyses, we will produce a thorough descriptive profile of the sample, characterising both the dropout and missing data. There are no interim analyses planned. Statisticians will not be blinded.

#### Baseline comparisons

Participant characteristics at baseline will be presented by treatment group, and statistical tests will be conducted to verify that randomisation was successful. Discrete variables will be summarised by frequencies and percentages, and baseline group differences tested using chi-square tests. Continuous variables will be summarised using mean (standard deviation) or median (interquartile range) and baseline group differences, tested using independent *t* tests or Wilcoxon signed-rank tests for variables that evidence non-normal distributions. If there are significant differences in any participant characteristics at baseline, the variable(s) will be included as covariates in the final analyses.

#### Aim 1

Aim 1 is to test the efficacy of CBT+ compared to TAU+ for improving symptoms of sleep disturbance. Aim 1 will be tested using LGMs. LGMs will be estimated with an intercept and two linear slopes, representing a piecewise model. Slope 1 will have loadings constrained to 0, 0.5, 1.0 and 1.0 for weeks 0, 3, 6 and 12, respectively, capturing the linear change from baseline (week 0) to post-intervention (week 6). Slope 2 will have loadings constrained to 0, 0, 0 and 1.0 for weeks 0, 3, 6 and 12, respectively, allowing a different slope from post-intervention (week 6) to follow-up (week 12) compared to from baseline (week 0) to post-intervention (week 6). The means and variances of the intercept and slope factors will be freely estimated (corresponding to random effects in linear mixed models) and the intercept and slope covariances will be estimated. The residual variance will be constrained to equality across time and residuals assumed uncorrelated, corresponding to an independent, homogenous residual structure. Intercepts of indicators will be constrained to 0 to allow estimation of the latent random intercept mean.

There are two stratification factors: screening ISI (≤7, ≥8) and cancer stage (≤2, ≥3). These factors will be crossed, creating four groups: early-stage, low ISI; advanced-stage, low ISI; early-stage, high ISI; and advanced-stage, high ISI. Dummy codes will be created for each strata, with early-stage, low ISI treated as the reference group. These dummy codes will be included as covariates to adjust for their effect on the random intercept following recommendations that stratification factors be adjusted for in analyses of randomised controlled trials [76, 77].

Treatment effects will be evaluated by creating a dummy code (0 = TAU+, 1 = CBT+). This dummy code will be entered as a predictor of the intercept, slope 1 and slope 2. However, treatment factors will be constrained to 0 for the intercept, to implement so-called constrained longitudinal data analysis, which studies show provides a more accurate estimate of treatment effects from randomised controlled trials with repeated measures [78, 79].

The primary trial results will be the effect of treatment on slope 1 (i.e. from week 0 to week 6). This interaction directly tests whether the change in primary outcomes over time is different in the control and intervention arm. Effect sizes of the group difference at each time point also will be calculated.

A similar process will be followed for sleep efficiency; however, a continuous time, linear model will be estimated using a mixed effects model in Mplus because up to 42 days of sleep efficiency are collected. A piecewise model is not needed as sleep diary data are not collected at follow-up due to burden. Sleep efficiency may not follow a normal distribution. If the assumption of normality is violated, significance tests and confidence intervals will be based on non-parametric bootstrapping.

#### Aim 2

Aim 2 is to test the efficacy of CBT+ versus TAU+ for psychological outcomes. Aim 2 will be tested identically to aim 1, but using psychological outcomes in place of the sleep and fatigue outcomes.

#### Aim 3

Aim 3 is to explore potential mechanisms of CBT+ and moderators of the intervention efficacy. Mechanisms (i.e. pre-sleep arousal, beliefs and attitudes about sleep, vulnerability to insomnia under stress and intrusive thoughts before bed) will be tested using mediation conducted in path analyses. Specifically, we will examine the effect of condition on change in mechanisms (for example, pre-sleep arousal) from baseline to treatment mid-point, and test whether the change in mechanisms from baseline to treatment mid-point accounts for the condition effects on change in outcomes (for example, ISI) from baseline to post-treatment and follow-up. Statistical mediation will be determined by evaluating the indirect effects, calculated as the product of the paths from condition to change in mechanisms and from change in mechanisms to change in outcomes. Bootstrapping will be used to estimate the confidence interval for indirect effects and their statistical significance. Given the modest sample size, analyses will be conducted for each mechanism and outcome individually.

Treatment moderators (i.e. pain, chronotype, perceived credibility and expectations of the intervention, and adherence to the intervention protocol) will be evaluated by modifying the primary linear mixed effects analyses from aims 1 and 2 to include a condition × moderator interaction, along with all lower order effects predicting slope 1 and slope 2 of the piecewise model. Individual moderators will be tested in separate models.

### Data storage and confidentiality

All participants will receive a random identification number which will be used to link data from the different assessments. Coding all participant data with a unique identification number will minimise the risk of loss of confidentiality. The only dataset with participant identifier information will be the participant tracking system used to follow-up and contact women. All other datasets will label participant records with a unique study number and be stripped of other identifying information; specifically, clinical data will not reside with identifying data. Survey data will be kept in locked files or password-protected data files. All results will be described in aggregate without identification of individual women. Study investigators will have access to the final dataset.

### Ethics approval and safety monitoring

The study has received ethics approval from the PMCC Human Research ethics committee, protocol number 17/159. Governance and oversight of the trial will be monitored by two committees, the Steering Committee and the Data and Safety Monitoring Board (DSMB). The Steering Committee comprises the study investigators and a consumer representative. The DSMB comprises two independent researchers, a consumer representative, and two members of the study investigator team. The DSMB will be responsible for monitoring participant safety, data quality, and implementation of the protocol. If any women report severe symptoms of sleep disturbance, depression, or anxiety, we will inform their clinical team and recommend that they seek a referral to a sleep specialist or clinical psychologist. Any women with previously undiagnosed severe psychiatric conditions identified during screening will also be informed and recommended to seek a referral to psychiatry or clinical psychology. A description of all undesirable and unanticipated events during the intervention phase (adverse events) will be recorded on Qualtrics. As this trial is minimally funded, there is no independent audit. Any protocol modifications will be communicated to the Human Research ethics committee and participants by the study team. No serious harm is expected as a result of the trial. However, if harm does occur, the primary sponsor and Monash University insurance will cover care for research participants.

#### Dissemination plan

The results of this study will be disseminated through peer-reviewed scientific publications and presentation at conferences. Three publications are anticipated: one paper reporting primary and secondary outcomes, a second paper focusing on mechanisms of treatment efficacy, and a third on moderators of treatment efficacy. Members of the study team who provide substantive contributions to the design, conduct and reporting of the study will be authors on these and any other unplanned publications based on the study data.

The full protocol and statistical code for data management and all primary analyses will be publicly available through Monash Figshare. A DOI has been reserved and the full study protocol including consent forms will be made available at 10.26180/5ccba90aae301 once the protocol paper is published. A DOI also has been reserved for statistical code for data management and primary analyses, which will become available once completed at 10.26180/5ccbaafb5262e.

## Discussion

This paper outlines the protocol for the development and evaluation of a novel intervention designed to improve the sleep of women sleep during chemotherapy treatment for BC. The results from this trial will advance the knowledge in this field and may have important clinical implications for how best to treat sleep disturbance and insomnia in this population.

The present trial is one of the first to target sleep complaints among women with BC currently undergoing chemotherapy treatment. Despite rates of sleep disturbance in this population being highest around the time of diagnosis and during treatment [80], most previous intervention studies have been carried out in women who have completed primary treatment for BC. The earlier treatment of sleep complaints is advantageous as it 1) may assist women by improving sleep during cancer treatment, and 2) provides women with evidence-based strategies earlier on in their treatment trajectory which they can continue to use after cancer treatment and into survivorship.

The intervention design is unique and was carefully considered to address the multiple causal factors for sleep disturbance in this population, while also being easily accessible and of minimal burden to participants. Furthermore, the largely email-based format of the CBT+ intervention allows for relatively easy dissemination of the programme should it be found to be effective. Clinical trials typically have low participation rates. One large randomised controlled trial that systematically approached women for a sleep intervention during treatment for BC had a 22% rate of consent to the randomised controlled trial following screening [32]. Another randomised controlled trial of people who had finished primary cancer treatment contacted 2531 people, of whom 327 (12.9%) expressed interest and 250 were eligible. Of eligible people who expressed interest in that study, 52.8% agreed to screening and random assignment [81]. In the current trial, 47.2% of women who were eligible and approached consented. This relatively high consent rate, particularly considering that women were undergoing chemotherapy and none had expressed prior interest nor been referred by their physicians, provides evidence supporting the intervention design and the feasibility for treating sleep disturbance during rather than after chemotherapy treatment. Furthermore, reasons for declining to participate have predominantly been due to women not being interested in being involved in research studies in general, or women already being involved in other studies and not wishing to participate in this one as well, rather than it being too burdensome an experience. Rates of recruitment and consent also have confirmed the prevalence of sleep disturbance in this population, with only 33 women of the 219 approached reporting no sleep complaints.

BLT and CBT impact on sleep through different mechanisms. Therefore, the combination of these intervention components should theoretically increase the magnitude of positive outcomes for participants compared to previous studies that have only evaluated CBT or BLT. If this preliminary study shows positive effects for women in the CBT+ group, a further study would be warranted with a larger sample size to evaluate the efficacy of the combined intervention in comparison to individual components (CBT or BLT). Analyses of potential mechanisms and moderators of treatment outcomes also will inform future research and allow for subsequent refinement and tailoring of the CBT+ intervention programme. A future goal for research in this field is to incorporate the treatment of sleep complaints into routine medical care for cancer patients.

## Trial status

Recruitment commenced in July 2018 and was completed in October 2019. One hundred and one women have been recruited and randomised to date (see Fig. 1). It is anticipated that all 3-month follow-up data will be collected by January 2020. This is protocol version number 7, 18 November 2019.

## Data Availability

Data sharing is not applicable to this article as no datasets were generated or analysed during the current study. However, once the trial is complete, data for the overall trial will be available from the study team by contacting the corresponding author on reasonable request.
